# Claudin3 is localized outside the tight junctions in human carcinomas

**DOI:** 10.18632/oncotarget.24858

**Published:** 2018-04-06

**Authors:** Michela Corsini, Antonella Ravaggi, Franco Odicino, Alessandro Davide Santin, Cosetta Ravelli, Marco Presta, Chiara Romani, Stefania Mitola

**Affiliations:** ^1^ Department of Molecular and Translational Medicine, University of Brescia, Brescia, Italy; ^2^ Department of Obstetrics and Gynecology, Division of Gynecologic Oncology, 'Angelo Nocivelli' Institute of Molecular Medicine, University of Brescia, Brescia, Italy; ^3^ Department of Obstetrics, Gynecology and Reproductive Sciences, Yale University School of Medicine, New Haven, CT, USA; ^4^ Department of Molecular and Translational Medicine, Laboratory for Preventive e Personalized Medicine, University of Brescia, Brescia, Italy

**Keywords:** claudin3, tight junction, ovarian cancer, uterine cancer

## Abstract

Claudin3 is an integral component of the tight junction proteins in polarized epithelia. The expression of claudin3 was assessed in epithelial-derived tumors using Oncomine database. To determine the gene alteration during carcinogenesis, copy number alterations and mutations of claudin3 were evaluated using cBioPortal database. Claudin3 is overexpressed in several tumors including gynecological, bladder, breast and prostate carcinomas. 38% of the 163 evaluated studies show mutations and/or amplification of claudin3. 3D reconstruction of tissue samples following immunofluorescence analysis clearly demonstrated that, unlike in healthy tissues, claudin3 is mislocalized and unengaged in the formation of tight junction in tumor samples. These data strongly support the evaluation of unengaged claudin3 as a target for the development of novel diagnostic probes, optical approaches for real time detection of tumoral tissues during surgery, and target therapeutic drugs.

## INTRODUCTION

Claudins belong to a large family of integral membrane proteins stably integrated within the tight junctions (TJ), at the apical end of the lateral membrane of epithelial cells. The claudin-based junctions are not static and undergo continuous molecular remodeling becoming a cell signaling component involved in the establishment and maintenance of cell polarity, sealing the intercellular space between adjacent cells and regulating the solute movement across epithelial sheets [1]. The expression pattern of the claudins is usually tissue specific; however, most tissues express multiple claudins that can interact in either a homotypic or heterotypic fashion to form the TJ strand.

Claudins also have functions in receiving environmental cues and transmitting signals inside cells, supporting cell growth and proliferation [2]. Indeed, the cytoplasmic C-terminal PDZ binding domain directly interacts with cytoplasmic protein ZO-1, −2 and −3, connecting claudins to the actin cytoskeleton and the signaling pathways of the cell [3].

The majority of tumors are of epithelial origin and therefore, similar to non-transformed epithelial cells, quite immobile. However, tumor cells may increase their mobility by the process of epithelial-to-mesenchymal transition (EMT). Epithelial cells down-regulate some epithelial cell-specific genes such as E-cadherin, alter the cell-cell contact and up-regulate some mesenchymal genes including *SNAIL*, *TWIST* and *SLUG*, which in particular promote migration and tolerance to a novel environment. Accordingly, there has been a description of alteration of TJ integrity and the down-regulation of different claudins, including claudin1 and claudin7, in a variety of epithelial tumors such as breast, esophagus and gastric cancers [4–6]. A claudin-low molecular subtype of breast cancer has been described with a concomitant upregulation of several EMT markers and an enrichment in stem cell features. This molecular subtype of breast malignancies is associated with poor prognosis in patients with high-grade invasive ductal carcinomas [7].

Surprisingly, the up-regulation of claudin3 and 4 in ovarian cancers [8], in uterine serous carcinomas [9], in colorectal cancers [10] and in breast cancers [11] has also been associated to tumorigenesis, though the role of the overexpressed claudins remains largely unexplained.

Alterations in the expression or epigenetic modulation of claudins affect apoptotic sensitivity to a number of apoptogens, invasiveness, and tumorigenicity in various cancer cells. It is noteworthy that dysregulated claudins directly result in several distinct abnormalities of tissue physiology.

The alteration of cell-cell contact leads to the loss of cell polarity, to the tissue disorganization and to the exposure of a number of extracellular signals such as those from growth factors. Also, in the absence of the apical-basal polarity, epithelial cells that receive growth signals not only in the apical domain tend to proliferate by an out-of-plane division promoted by the mis-orientation of the mitotic axis [12]. Thus, the loss of polarity in tumor tissues might suggest an altered localization of claudin in tumor cells.

We recently developed the IgGH6 antibody against the minor extracellular domain of claudin3, which is physiologically engaged in the cell-cell contact in the TJ. IgGH6 recognizes a subset of claudin3 non-engaged in TJ formation [13]. Since TJ proteins serve as a physical barrier regulating paracellular permeability, their alteration may alter not only tumor infiltration but also tumor cell metastatization. This unengaged or mislocalized claudin3, if it exists *in vivo*, might represent not only a good biomarker of cancer progression but also a good target for the development of tumor directed therapy.

Here we analyzed the alterations of claudin3 in terms of expression and localization in different epithelial-derived human tumors. The expression and genomic alterations were examined in public Oncomine and cBioPortal database, while immunofluorescence analysis using z-stack tool was used to unequivocally demonstrate that claudin3 in tumors, (as different authors have speculated), is not confined to TJ but is relocated along the surface of the cells. The alteration of claudin3 localization is not due to its expression levels but it is an intrinsic characteristic of the neoplasia.

## RESULTS

### Claudin3 expression and genomic alterations in epithelial-derived tumors

**Figure 1:**
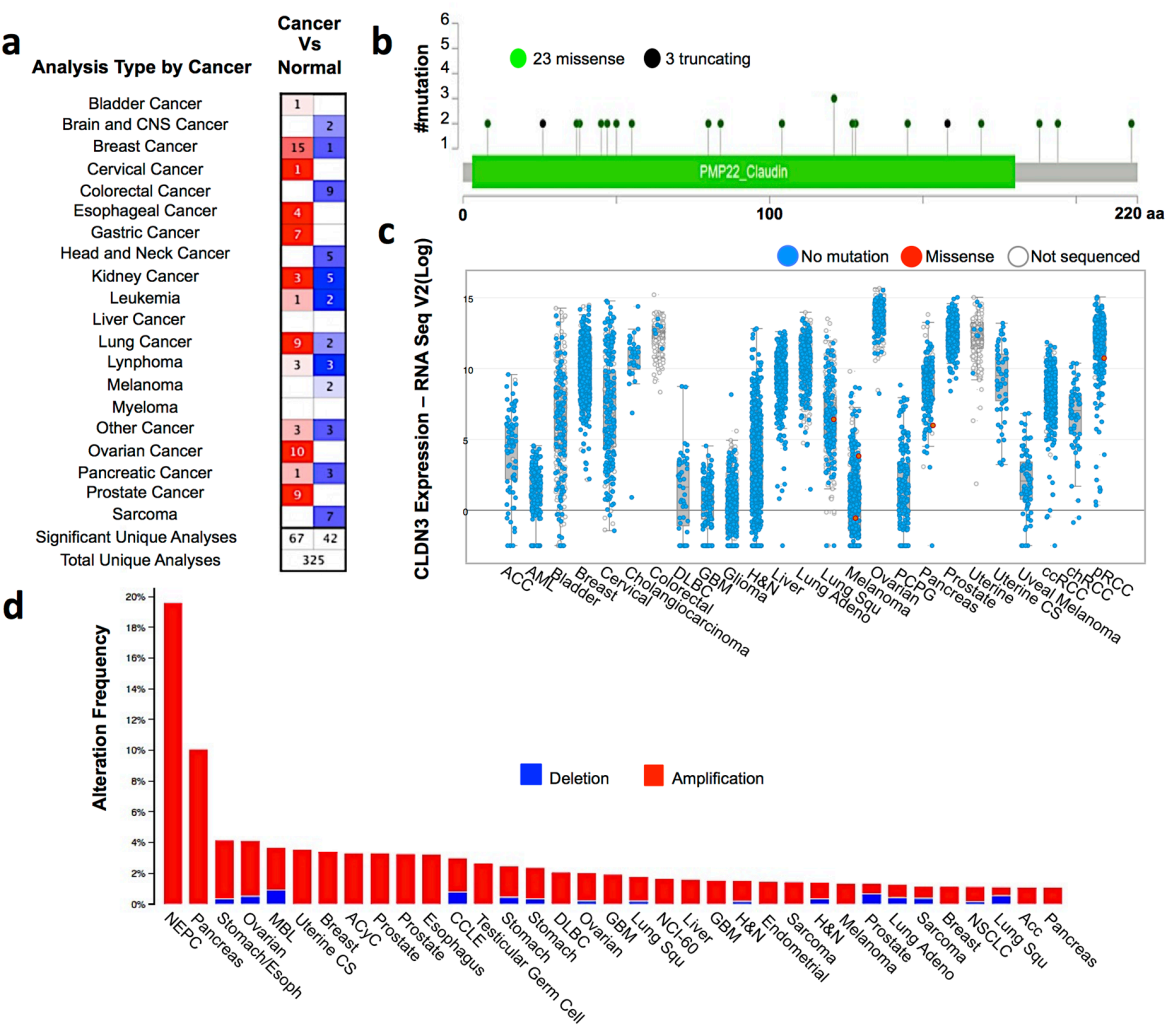
Claudin3 is altered in tumors **(a)** The comparison indicated the number of datasets with claudin3 mRNA overexpression (left column, red) and under expression (right column, blue) in cancer versus normal tissue. The threshold was designed with following parameters: *p*-value of 0,005, fold change of 1.5, and gene ranking of 10% Oncomine (www.oncomine.com). **(b)** claudin3 frequency mutations. **(c)** correlation between claudin3 expression level and genomic mutations. **(d)** alteration frequency of claudin3 signature was determined using the cBioPortal (http://www.cbioportal.org). The alteration frequency included deletions (blue) and amplification (red).

An altered claudin3 expression has been reported in different tumors. Comparative gene expression analysis between tumor and normal tissues using the cancer microarray database and the data mining platform of Oncomine revealed that claudin3 is overexpressed in breast, esophageal, gastric, ovarian, lung and prostate cancers. The threshold was designated according to the following values: p-value <0.005, fold change 1.5, and top gene ranks 10% (Figure 1a). We next explored the genomic alterations of claudin3 by examining the mutational data publicly available in the cBioPortal database. CBioPortal collects all information on gene alteration in frequency, including deletions, amplification or multiple alterations, and on gene mutations of 681 cancer samples. This analysis identified only 23 low frequency missense and 3 truncating mutations in claudin3 sequence in the 163 studies analyzed (Figure 1b). Mutations mainly occurred in the primary central nervous system and lymphoma, which exerts the higher mutational profile. Overall, 10% of the examined samples contain missense mutations in claudin3 sequence (Figure 1c). Figure 1c correlates the expression level of claudin3 with missense mutations, suggesting that there is no relationship between mutation and overexpression. Claudin3 amplification was described in most analyzed tumors with a variable percentage, ranging from about 18% in neuroendocrine prostate cancer to 1% in other samples (Figure 1d).

### Claudin3 expression in gynecological tumors

**Figure 2:**
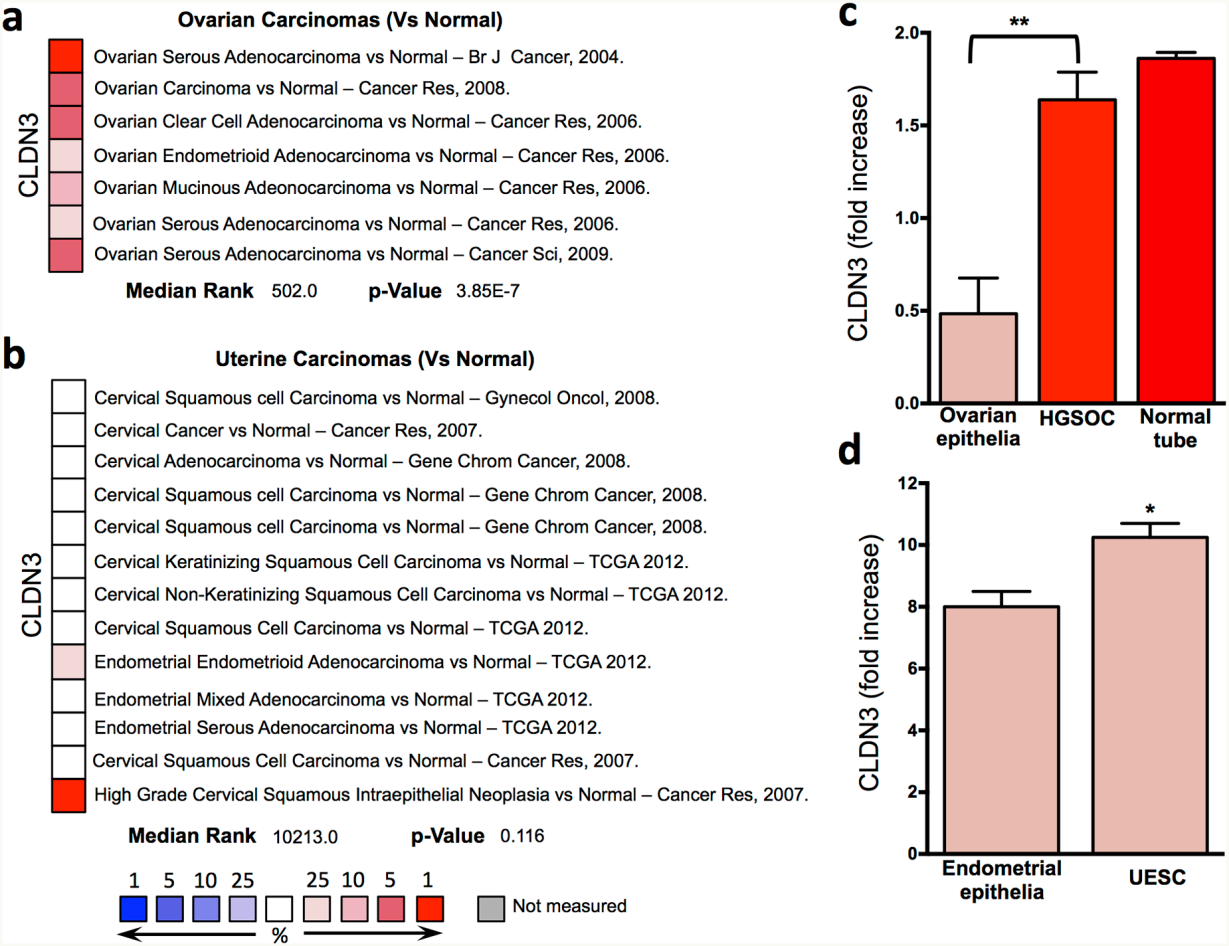
Claudin3 in gynecological tumors **(a-b)** claudin3 analysis in different ovarian and uterine cancer histotype available in Oncomine datasets. **(c-d)** claudin3 expression evaluated by qRT-PCR in fresh frozen biopsies of ovarian and endometrial serous carcinomas and fallopian tube. (^**^P<0.003, ^*^P <0.06 unpaired t test, n=5).

When we analyzed in more detail the available datasets of gynecological tumors, we observed the over-expression of claudin3 in different histotypes of epithelial ovarian cancer, including serous, endometrioid, clear cell and mucinous (Figure 2a), while no significant changes was highlighted in uterine carcinomas including endometrioid and serous adenocarcinomas (Figure 2b).

Claudin3 expression was verified in fresh frozen biopsies collected in our tissue bank of serous ovarian and endometrial carcinomas. In High Grade Serous Ovarian Carcinoma (HGSOC) the expression of claudin3 is 4 fold higher than in healthy ovarian epithelia, where claudin3 is almost undetectable (Figure 2c). Whereas no significant alteration in claudin3 expression can be appreciated if, following the recently idea that HGSOC arise from fallopian tube, we compare HGSOC with healthy tube (Figure 2c). A slight increase is observed in Uterine Endometrial Serous Carcinoma (UESC) samples compared with normal endometrial epithelia (Figure 2d).

### Claudin3 is not confined to TJ in tumor samples

**Figure 3:**
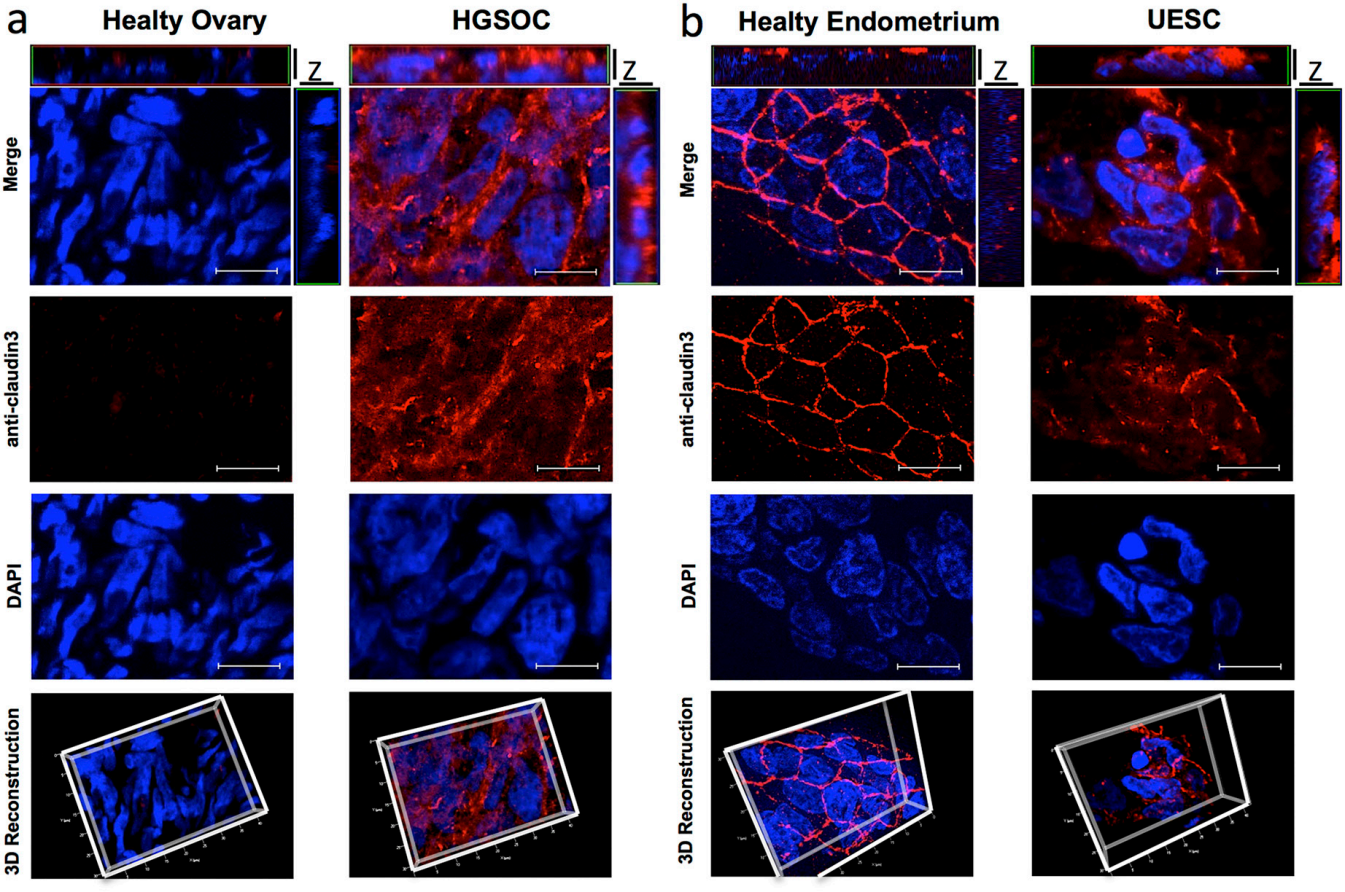
Claudin3 is out of tight junctions in in tumor samples 20 μm section of fresh frozen biopsies of healthy **(a)** and tumor **(b)** ovarian and uterine endometrial tissues were immunostained with a commercial anti-claudin3 antibody followed by anti-rabbit Alexa594. Nuclei are counterstained with DAPI. Z-stack images were recorded using a Zeiss Axiovert 200M epifluorescence microscope equipped with a Plan-Apochromat 63x/1.4 NA oil objective and ApoTome system. For 3D reconstructions and orthogonal projections, Z-stack images were elaborated with AxioVision Inside4D. Scale bar: 20 μm.

**Figure 4:**
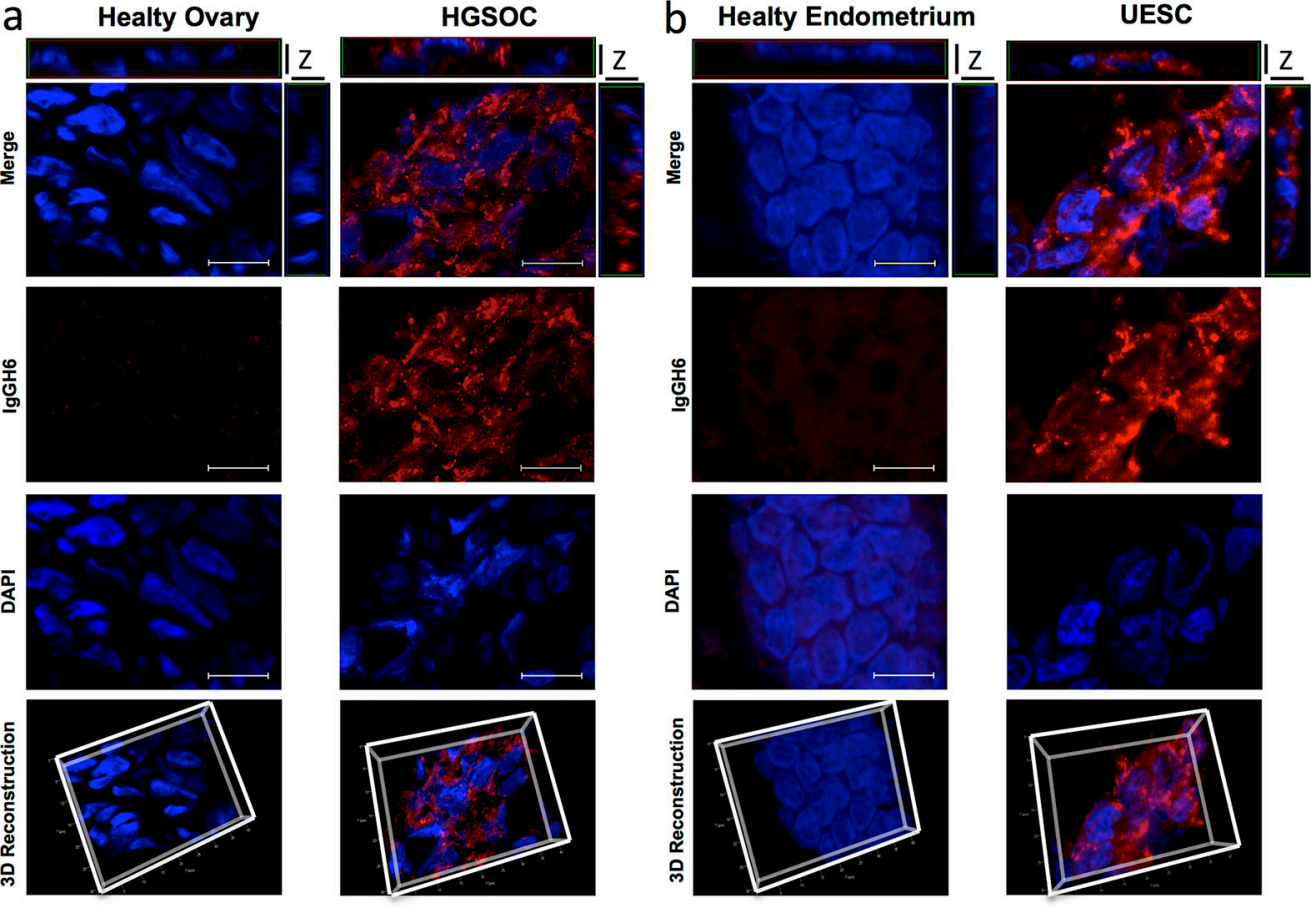
Mis-localized Claudin3 is a suitable target for tumor identification **(a-b)** 20 μm section of fresh frozen biopsies of healthy and tumor ovarian and uterine endometrial tissues were immunostained with an IgGH6 antibody against minor extracellular domain of claudin3 followed by anti-human Alexa594. Nuclei are counterstained with DAPI. Z-stack images were recorded using a Zeiss Axiovert 200M epifluorescence microscope equipped with a Plan-Apochromat 63x/1.4 NA oil objective and ApoTome system. For 3D reconstructions and orthogonal projections, Z-stack images were elaborated with AxioVision Inside4D. Scale bar: 20 μm.

Several authors speculated that over-expressed claudins are not only confined to cell-cell contacts and may lead to a loss of epithelial cell polarization. To verify whether the expression alteration of claudin3 affects its localization on cell membrane, we incubated a 20 μm section of fresh frozen biopsies of healthy and tumor tissues with the commercial antibody against the intracellular portion of claudin3. Of note, this antibody allows the staining of all expressed claudin3. As expected, immunofluorescent analysis confirms that healthy ovary tissue does not express detectable levels of claudin3 (Figure 3a) whereas 3D reconstruction of Z stack analysis of healthy endometrium shows a dense staining at the apical lateral side of normal epithelium, with claudin3 homogeneously distributed along the cell-cell contact throughout the plasma membrane (Figure 3b). Then, to characterize the effect of upregulation of claudin3 in tumor, we performed a 3D reconstruction of claudin3 localization in all HGSOC and UESC samples. Immunofluorescence does not allow to appreciate claudin3 over-expression, but its disordered distribution on cell membrane certainly does. The membrane expression of claudin3 is also demonstrated by co-staining with anti claudin3 and WGA-lectin of 3D culture of UESC-derived cells (Supplementary Figure 1). In tumor sections claudin3 is unevenly distributed and tight junctions are not clearly identified in the apical lateral side of cell-cell contact (Figure 3a and 3b). We could not exclude that mislocalized claudin3 are engaged in a cell-cell contact structures. To address this point, we re-analysed the same tissue samples using IgGH6 antibody, recently developed in our laboratory. IgGH6 recognizes the minor extracellular domain of claudin3 and labels only the unengaged claudin3 [13]. Figure 4 shows that in normal tissues IgGH6 antibody does not recognize claudin3 suggesting, in accordance with our previous results, that all claudin3 expressed on cell membrane are committed to the formation of tight junctions. In tumor samples, on the contrary, 3D tissue reconstruction clearly reveals that claudin3 is mislocalized and not involved in cell-cell contacts both in HGSOC and UESC samples (Figure 4). These data suggest that the up-regulation of claudin3 is not necessary for its mislocalization. Indeed, IgGH6 antibody recognizes claudin3 also in tumoral tissue, such as UESC, in which there is not a clear transcriptional up-regulation.

All these data, in agreement with the previous observations, support the idea that the dysregulation of claudin3 expression in tumoral tissue might play a role in the pathogenesis of cancer.

## DISCUSSION

In this paper we analyzed the expression and localization of claudin3 in different healthy tissue and human tumor samples, using a combined approach based on *in-silico* evaluation of claudin3 gene expression and genomic alterations, RT-qPCR and immunofluorescence staining on fresh-frozen specimens. Our analysis, performed on a large dataset of human carcinomas and healthy tissues revealed a substantial dysregulation of claudin3 expression in all evaluated tumors and confirmed that the observed differential expression is tissue-specific and strictly dependent on cancer type [14]. According to the cBioPortal database examination, there was no direct correlation between claudin3 expression and its genomic alterations in terms of deletions, amplifications or single mutations, suggesting that claudin3 expression changes in tumors were not influenced by the gene mutational status. Importantly, we unquestionably demonstrated that in epithelial tumors claudin3 appears heterogeneously distributed outside the tight junctions, and this is apparently unrelated to its expression level. These altered localization with the presence of a subset of claudins outside the TJ, has been hypothesized as a consequence of the dis-regulation of the mitotic axis occurring in malignant proliferating cells, causing out-of-plane division of tumor cells and aberrant exposure of junctional components [12]. This phenomenon, however, has never been clearly addressed due to the lack of functional antibodies specific for claudin extracellular domains. Here we confirmed the claudin3 expression in fresh samples collected in our Institute, focusing on ovarian and endometrial serous carcinomas where claudin3 has been consistently reported as potential cancer biomarker and therapeutic target by our group and others [15–17]. Although public datasets used in our study refereed HGSOC biogenesis to ovarian epithelium, a new paradigm on the pathogenesis of HGSOC is emerging, which identifies the fallopian tube epithelium as a putative site of origin of this tumor [18]. Since we can not exclude this hypothesis, we also compared claudin3 expression in HGSOC with tube surface epithelium. Preliminary results show significant reduction in claudin3 expression (Romani C, unpublished results). Ovarian surface epithelium is a modified mesothelium *in continuum* with the mesothelial lining of the pelvic organs, and embryological distinct from Mullerian epithelia [19]. Not surprisingly claudin3 is almost undetectable in healthy ovarian samples but abundantly expresses and properly engages in a cell-cell contact structures in Mullerian-derived tube epithelium. Of note here we show claudin localization in ovarian and uterine tumors but similar results were obtained also in other epithelial tumors including colon carcinomas (Corsini M. unpublish data).

The importance of expression of claudins and their localization in diagnostic is supported by the increasing number of papers suggesting a relation between claudin expression and tumor outcome. A claudin^low^ breast cancer subtype, which expressed low level of claudin4, claudin7 and claudin3, has been identified using human tumor database. In contrast to the basal-like subtype, claudin^low^ tumors are more enriched in epithelial-to-mesenchymal transition features, immune system responses, and stem cell-associated biological processes [7]. Importantly, claudin^low^ tumors show some chemotherapy sensitivity and have a poor prognosis. Starting from these data, Prat et al. developed a genomic differentiation predictor for the classification of breast tumors. Moreover, an invasive ductal breast carcinoma subgroups has been characterized by immunohistochemistry analysis by the expression of Ki-67, cytokeratins (CK5 and CK18) and claudin7 [20]. Also the expression of claudin11 has been suggested as biomarker for advanced stage of cutaneous squamous carcinoma [21]. On these basis, we can speculate that the differential expression of claudins may reflect the distinct stages of tumor development and differentiation. A panel of antibodies against claudins could be used in diagnostic to complete the tumor characterization and to help the therapeutically choice. In this regard, the IgGH6 may be able to distinguish the expression of the claudin3 of healthy tissues, in which it is located in the TJ, from the tumor ones.

In summary, we can conclude that the transcriptional up-regulation of claudin3 is not related to its mislocalization and that the relocalization of claudins out of the TJ is in agreement with the loss of polarized morphology that characterizes epithelial cells undergoing neoplastic transformation.

Staining with IgGH6 human antibody, whose binding epitope is located within the minor ectodomain of claudin3, clearly proves the presence of extra-junctional claudin3 outside the cell-cell contact in transformed epithelia. Minor ectodomain is engaged in claudin-claudin homotypic interactions and becomes accessible only in tumor cells characterized by an alterate cell-cell junctions. This unengaged claudin3 represents a potential target both for antibody-based diagnostic probes.

To our knowledge, IgGH6 is the first molecule able to bind exclusively unengaged claudin3. IgGH6 represents an unvaluable tool to assess claudin3 mislocalization on epithelial-derived cancer cells.

## MATERIALS AND METHODS

### Oncomine and cBioPortal databases analysis

The public sites Oncomine (https://www.oncomine.org) and cBioPortal (www.cbioportal.org) were used respectively for the analysis of expression and genomic alterations of claudin3. These provided access to data from more than 5,000 tumor samples from 163 cancer studies in the TCGA pipeline.

### Tissue samples

Tissue samples were collected from patients undergoing surgery at the Division of Obstetric and Gynecology, ASST-Spedali Civili (Brescia, Italy), under a protocol approved by the institutional review board (study reference number: NP1284). The study was performed following the Declaration of Helsinki set of principles and was approved by the institutional Research Review Board-Ethic Committe. Written informed consent was obtained from each patient before collecting tissue samples. Tumor specimens of 5 uterine endometrial serous carcinoma (UESC) and of 5 high grade serous ovarian carcinoma (HGSOC) were collected at the time of primary surgery and snap frozen in liquid nitrogen. Normal endometrial (NE) tissue samples were obtained from 5 patients undergoing hysterectomy for benign pathologies. Five Normal ovarian (NO) samples were collected by scraping the surface epithelium of normal ovaries [22].

### Quantification of claudin3 mRNA by RT-qPCR

Samples containing at least 70% of tumor cells were processed for RNA extraction using TRIzol Reagent (Invitrogen, Carlsbad, CA) followed by RNeasy MiniElute Cleanup kit (Qiagen). cDNA was generated from total RNA using the SuperScriptII reverse transcriptase (Invitrogen) [23]. Quantitative PCR was performed with a Biorad CFX96 Real-Time PCR Detection System using iQ™ SYBR Green Supermix (Biorad, Hercules, CA). Data were recorded using Bio-Rad CFX Manager software (BioRad). Relative expression ratios were calculated by use of Pfaffl equation and Relative Expression Software Tool. The 2^−ΔΔCt^ method was applied to calculate changes in claudin3 expression. The mRNA expression levels of claudin3 were normalized to the geometric mean of HPRT1 and PPIA transcript levels. TaqMan Gene Expression Assay for claudin3 was obtained from Applied Biosystems as Assay-on-Demand product (ID: Hs00265816_s1). HPRT1 (primers for/rev: CTGGAAAGAATGTCTTGATTGTG/GACCTTGACCATCTTTGGATTA and probe ACTGCC TGACCAAGGAAAGCAAAGTCT) and PPIA (primers for/rev: GAGGAAAACCGTGTACTATTAGC/GGGAC CTTGTCTGCAAAC and probe CCACCGTGTTCTTCG ACATTGCCGT) assays were designed with Beacon Designer software (Premier Biosoft, Palo Alto, CA, USA).

### Immunofluorescence staining

Snap frozen tissue sections were fixed in 3% paraformaldehyde/2% sucrose in PBS, permeabilized with 0.5% Triton-X100, and saturated with goat serum in PBS. Samples were incubated with anti-claudin3 (Invitrogen) or with IgGH6 anti-claudin3 [13] over-night at 4°C followed by Alexa Fluor 594 anti-rabbit IgG (Molecular Probes, Eugene, OR). Cells membranes were stained with WGA lectin-Alexa Fluor 488 (Molecular Probes, Eugene, OR) and nuclei were counterstained with 4’,6-diamidino,2-phenylindole (DAPI, Sigma). Samples were analyzed using Zeiss Axiovert 200M epifluorescence microscope equipped with Apotome system, Plan-Neofluar 20x/0.5 NA and Plan-Apochromat 63x/1.4 NA oil objectives. Z-stack images were elaborated with AxioVision Inside4D module (Carl Zeiss) [24].

## SUPPLEMENTARY MATERIALS FIGURE



## Abbreviations

TJ: Tight junction
EMT: epithelial-to-mesenchymal transition
UESC: uterine endometrial serous carcinoma
HGSOC: high grade serous ovarian carcinoma
NE: Normal endometrial
NO: Normal ovarian
HPRT1: Hypoxanthine Phosphoribosyltransferase 1
PPIA: Peptidylprolyl isomerase A
DAPI: 4’,6-diamidino,2- phenylindole.
