# Supplementary material for: Claudin3 is localized outside the tight junctions in human carcinomas
Source: Oncotarget. 2018 Apr 6;9(26):18446–53. doi: 10.18632/oncotarget.24858 (PMC5915084; doi:10.18632/oncotarget.24858)
Supplement: Supplementary file 1 [file oncotarget-09-18446-s001.pdf]

Claudin3 is localized outside the tight junctions in human carcinomas

SUPPLEMENTARY MATERIALS

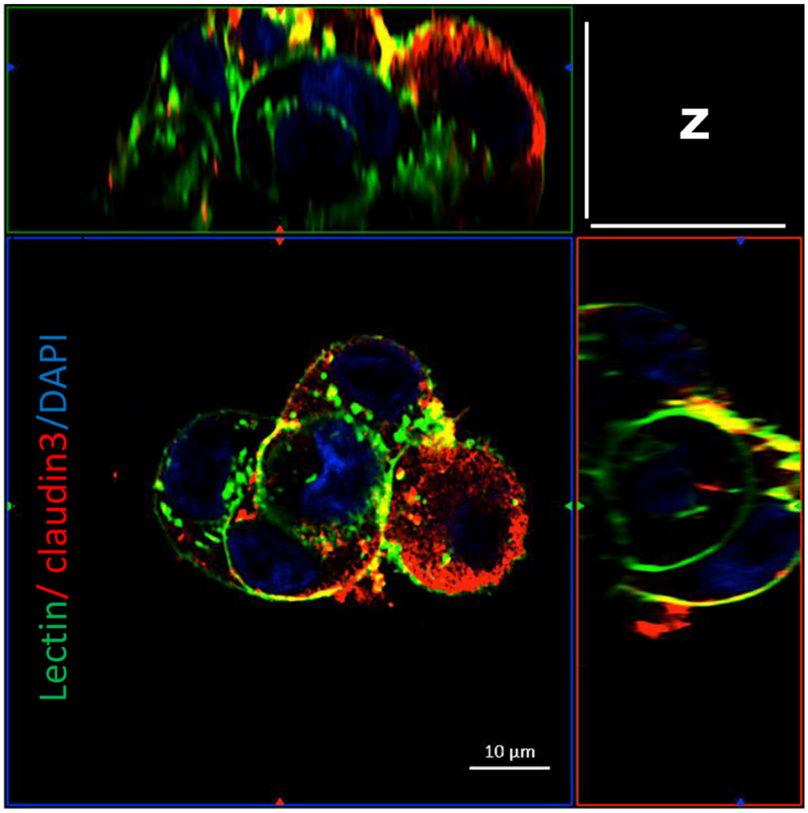

Supplementary Figure 1: Claudin3 is localized in plasma membrane. 3D cultures of UESC-derived cells were embedded in collagen gel and immunostained with a commercial anti-claudin3 antibody followed by anti-rabbit Alexa594. Cells membranes were labeled with Alexa488 conjugated WGA-lectin and nuclei were counterstained with DAPI. Z-stack images were collected with LSM510 Meta confocal microscope equipped with EC Plan-Neofluoar 40x/1.3 oil Dic. For orthogonal projection, z-stack images were elaborated with AxioVision Inside4D module (Carl Zeiss). Scale bar: 10 µm.
